# Supplementary material for: Unfolding and de-confounding: biologically meaningful causal inference from longitudinal multi-omic networks using METALICA
Source: mSystems. 2024 Sep 6;9(10):e01303-23. doi: 10.1128/msystems.01303-23 (PMC11494969; doi:10.1128/msystems.01303-23)
Supplement: Supplemental material — Organization of Metalica website, details on experiments, and settings of all parameters in experiments. [file msystems.01303-23-s0001.pdf]

mSystems Submission Template

Supplement to "Unfolding and De-confounding: Biologically meaningful causal inference from longitudinal multi-omic networks using METALICA"

Daniel Ruiz-Perez,[a] Isabella Gimon,[a] Musfiqur Sazal,[a] Kalai Mathee,[b,c] Giri Narasimhan[a,c]

Compiled June 12, 2024
This is a draft manuscript, pre-submission

Address correspondence to Giri Narasimhan, giri@fiu.edu.

The supplemental website http://bit.ly/Metalica_Public is organized as follows. It has five subdirectories:

Code This contains source code and scripts used in the work. The files 'execut-eDBN.m', 'pycausal.py', and 'tigramite.py' are the ones used to learn the different networks. The files 'unrolling.R' and 'deconfounding.R' are the ones used to compute the unrolling and deconfounding results, respectively.

Data Each file contains a single omic data set, in either the aligned or unaligned formats, with one of five different constraint matrices (HEGTM, HEGTM_Skeleton, TM, TG, GM), and for both the Crohn's disease subset and the subset with all disease statuses are available here.

Networks All networks created in this work are made available here. They are all in GRAPHML format, with a naming convention explained below.

Unrolling All results from the unrolling approach are provided here in csv formats for each combination of parameters, as explained below. Note that we are using all the omic subsets in order to generate one combined file for the results of unrolling. The column headers of the file are as follows:

- T and T_i are the source and target taxon of the unrolling operation.
- Tx, G, and M represent the taxon, gene, or metabolite intermediary discovered by unrolling for the edge from T to T_i.
- $T \rightarrow (G|Tx)\_Bs$, $(T|G) \rightarrow M\_Bs$, $(M|G|Tx) \rightarrow T\_i\_Bs$, and $T \rightarrow T\_i\_Bs$ are bootstrap scores from unrolling of the row.
- $overallScore$ is the multiplication of the three bootstrap scores.
- $ChainInTTxTi$, $ChainInTGMTi$, $ChainInTGTi$, and $ChainInTMTi$ are booleans indicating whether that interaction also appears in the data subsets that are mentioned.

The files 'statsAllAugmented.csv' and 'overallAVGStatsForEachMethod.csv' are statistics for each network execution, and the overall statistics for all methods, respectively.

De-confounding All results from the de-confounding approach are provided here in csv formats for each combination of parameters, as explained below. Note that we are using all the omic subsets in order to generate one combined file for the results of de-confounding. The column headers of the file are as follows:

- $confounder$, $confoundedParent$, and $confoundedChild$ are the names of the three nodes involved in the confounding, and are therefore self-explanatory.

mSystems Submission Template

- $BootscoreConfounderP$, $BootscoreConfounderC$, and $BootscoreConfounded$ are the bootstrap scores of the nodes from the item above. $overalScore$ is the result of their product.
- $method$, $sourceLevel$, and $deconfoundedWith$ are the names of the network-learning method, the dataset where the confounded relationship was found, and the dataset used to de-confound it, respectively.

The additional files 'mostCommonConfounders.csv', 'overallDeconfoundingStats.csv', and 'overallDeconfoundingStatsByMethod.csv' contain the list of all confounders found in this experiment, ordered by the number of times they were found, and the overall statistics of the results.

Details on the networks created are given below.

DYNAMIC BAYESIAN NETWORKS COMPUTED USING PALM.

DBNs were learned using PALM for all subsets of the omics datasets mentioned in the manuscript ($\mathbb{T}$, $\mathbb{TG}$, $\mathbb{TM}$, $\mathbb{GM}$, $\mathbb{TGM}$), alignment option (yes, no), sampling rate (7 days), number of bootstrap repetitions (100), number of allowable parents ($\{3, 4, 5, 6\}$), and the different types of constraint matrix used (HEGTM, HEGTM_Skeleton, TM, TG, GM). Some combination of parameters yielded empty networks and were not saved, making a maximum of $5 \times 2 \times 1 \times 1 \times 4 \times 5 = 200$ potential networks.

The networks were then combined, averaging the regression coefficient (weight) of the edges as long as they appeared in at least 10% of the repetitions. Each edge was also labeled with the bootstrap score or support (proportion of times that edge appears). Each repetition was set to run independently on a separate processor using Matlab's Parallel Computing Toolbox. All the networks from these experiments are made available. The network files are named using the following convention:

$DBN\_\{DataSubset\}\_\{Dataset\}\_\{AlignmentOption\}\_sr\{SamplingRate\}d\_$
$nboots\_\{BootstrapRepetitions\}\_nParents[MaximumNumberOfParents]\_$
$matrix\{ConstraintMatrix\}\_s\{ComputationTime(seconds)\}.graphml$

CAUSAL NETWORKS USING THE TETRAD SUITE.

Networks computed with TETRAD that are made available were learned for each omics dataset ($\mathbb{T}$, $\mathbb{TG}$, $\mathbb{TM}$, $\mathbb{GM}$, $\mathbb{TGM}$), alignment option (yes, no), sampling rate (7 days), significance threshold $\alpha \in \{0.0001, 0.001\}$, number of bootstrap repetitions (10), the FisherZScore network score, type of CI test (PositiveCorr, FisherZ, DegenerateGaussianLRT, SemBIC), maximum number of parents (3), and the different types of constraint matrix used (HEGTM, HEGTM_Skeleton, TM, TG, GM). Some combination of parameters yielded empty networks and were not saved, which makes for a maximum of $5 \times 2 \times 1 \times 2 \times 1 \times 1 \times 4 \times 1 \times 5 = 400$ potential networks.

The files are named using the following convention:

$PyCausal\_\{DataSubset\}\_\{Dataset\}\_\{AlignmentOption\}\_sr\{SamplingRate\}d\_$
$a\{\alpha\}\_nboots\{BootstrapRepetitions\}\_score\{ScoreName\}\_test\{CITestName\}\_$
$nParents\{MaximumNumberOfParents\}\_matrixmatrixName\_$
$s\{ComputationTime(seconds)\}.graphml$

CAUSAL NETWORKS WITH TIGRAMITE.

Networks computed with Tigramite that are made available were learned for each omics dataset ($\mathbb{T}$, $\mathbb{TG}$, $\mathbb{TM}$, $\mathbb{GM}$, $\mathbb{TGM}$), alignment option (yes, no), sampling rate (7 days), bootstrap threshold (0.1), significance threshold values $\alpha \in \{0.0001, 0.001, 0.01, 0.1\}$, Tau max of 1, type of CI test (GPDC, CMIknn, ParCorr) (**?**), and the type of constraint matrix used (HEGTM, HEGTM_Skeleton, TM, TG, GM). Some combination of pa-

mSystems Submission Template

rameters yielded empty networks and were not saved, which makes for a maximum of $5 \times 2 \times 1 \times 1 \times 4 \times 1 \times 3 \times 5 = 600$ potential networks.

The files are named using the following convention:

$Tigramite\_\{DataSubset\}\_\{Dataset\}\_\{AlignmentOption\}\_sr\{SamplingRate\}d\_bs\_\{BootstrapLevel\}\_a\{\alpha\}\_ptreshold\{pvalueTreshold\}\_T\{TauMax\}\_test\{CITestName\}matrixName.matrix\_s\{ComputationTime(seconds)\}.graphml$
